# Machine learning and BP neural network revealed abnormal B cell infiltration predicts the survival of lung cancer patients

**DOI:** 10.3389/fonc.2022.882018

**Published:** 2022-10-11

**Authors:** Pinghua Tu, Xinjun Li, Lingli Cao, Minghua Zhong, Zhibin Xie, Zhanling Wu

**Affiliations:** Department of Respiratory and Critical Care Medicine, Xiaogan Hospital Affiliated to Wuhan University of Science and Technology, Xiaogan City, China

**Keywords:** random forest, back propagation (BP) neural network, COX regression, prognostic prediction, immune infiltration

## Abstract

FAM83A gene is related to the invasion and metastasis of various tumors. However, the abnormal immune cell infiltration associated with the gene is poorly understood in the pathogenesis and prognosis of NSCLC. Based on the TCGA and GEO databases, we used COX regression and machine learning algorithms (CIBERSORT, random forest, and back propagation neural network) to study the prognostic value of FAM83A and immune infiltration characteristics in NSCLC. High FAM83A expression was significantly associated with poor prognosis of NSCLC patients (p = 0.00016), and had excellent prognostic independence. At the same time, the expression level of FAM83A is significantly related to the T, N, and Stage. Subsequently, based on machine learing strategies, we found that the infiltration level of naive B cells was negatively correlated with the expression of FAM83A. The low infiltration of naive B cells was significantly related to the poor overall survival rate of NSCLC (p = 0.0072). In addition, Cox regression confirmed that FAM83A and naive B cells are risk factors for the prognosis of NSCLC patients. The nomogram combining FAM83A and naive B cells (C-index = 0.748) has a more accurate prognostic ability than the Stage (C-index = 0.651) system. Our analysis shows that abnormal infiltration of naive B cells associated with FAM83A is a key factor in the prognostic prediction of NSCLC patients.

## Introduction

Lung cancer is a common malignant tumor in clinical practice in the respiratory system, with high morbidity and mortality. Non-small cell lung cancer (NSCLC) accounts for around 80% to 85% of all lung cancer cases. (1). Currently, distant metastasis and recurrence are still a severe challenge to NSCLC undergoing surgery (2). Targeted drugs and immunotherapy have opened a new era of NSCLC treatment (3). However, only a fraction of patients benefit from immunotherapy (4). In addition, current prognostic biomarkers are insufficient to accurately assess NSCLC patients’ prognosis due to tumor cell heterogeneity and drug resistance (4, 5). As a result, developing potent and precise prognostic biomarkers is critical for improving NSCLC prognosis and tailored treatment.

On chromosome 8q24, the family with sequence similarity 83 member A (FAM83A) gene, is found. Initially, the authors identified FAM83A as a potential oncogenic gene through bioinformatics methods. Previous studies reported that FAM83A plays a role in promoting cancer in a variety of tumors (6). Lee et al. (7) revealed that FAM83A overexpression induced resistance to epidermal growth factor receptor-tyrosine kinase inhibitor (EGFR-TKI), leading to the breast cancer patients’ poor prognosis (8). Recently, Hu et al. (9) found that FAM83A may promote NSCLC tumorigenesis through the ERK and PI3K/Akt/mTOR pathways. However, studies have found that FAM83 family genes maybe a risk factor for the NSCLC patients survival (10). However, the abnormal infiltration of immune cells associated with FAM83A is poorly understood in the pathogenesis and prognosis of NSCLC. Studying the FAM83A immune infiltration may guide the treatment and prognosis of NSCLC.

The widely used machine learning algorithms enable analyzing large population gene sequencing or microarray data from a new perspective. Machine learning algorithms, including random forest, least absolute shrinkage and selection operator (LASSO), and the bio-inspired algorithm, back propagation neural network (BPNN) can avoid the risk of overfitting. And the biomarkers defined by machine learning algorithms have shown better performance in prognostic prediction than those developed through traditional statistical methods in the past decades (11). Therefore, the machine learning algorithms identified a biomarker related to the prognosis and immune infiltration of NSCLC.

The study first comprehensively analyzed the expression level, prognostic performance, and clinical relevance of FAM83A in NSCLC. Subsequently, we tested the prognostic independence of FAM83A. Next, we applied CIBERSORT deconvolution algorithm to determine immune cell infiltration in the NSCLC microenvironment. Finally, we used LASSO, random forest (RF) and BPNN algorithms to identify immune cells with abnormal infiltration associated with FAM83A.

## Materials and methods

### Data source

The cancer genome atlas (TCGA, https://cancergenome.nih.gov) database was used to download lung adenocarcinoma (LUAD) and lung squamous cell carcinoma (LUSC) expression data as well as clinical follow-up data. Then we kept the expression matrix of 20,531 genes containing 110 normal and 1019 tumor samples. At the same time, the mRNA expression of GSE37745 containing 196 samples and the corresponding clinical data are downloaded from the gene expression omnibus (GEO, https://www.ncbi.nlm.nih.gov/geo/) database. Finally, we normalized data and eliminated patients with a survival time ≤ 0 months.

### CIBERSORT analysis

We used the support vector machine (SVM) based CIBERSORT deconvolution algorithm to investigate the heterogeneity in the tumor immune microenvironment (12). Subsequently, we run 1000 permutations through the LM22 gene signature file to evaluate 22 immune cells’ infiltration scores.

### LASSO analysis

Based on FAM83A expression grouping(group by median), we used LASSO analysis to screen key immune cells from 22 immune cells. LASSO applied the L1 norm to punish the model for achieving constraints on the objective function (13).

The complexity of the model is controlled by λ. Specifically, the penalty of the linear model is positively correlated with λ. In this study, when we call rng(seed) to generate random numbers, the numbers generated each time are random. However, when we call and set the seed value in advance, the random number generated by rng(seed) will be the same.

### Random forest analysis

The main idea of random forest (RF) is to obtain a series of decision trees (14). The algorithm captures complex interactions to get a set of average features. The study uses the “randomForest” package to implement the training: (1) extract patient samples from the training data set TCGA as a random subset (2) use a random subset of predictors to grow each tree. Tree branches grow to the maximum without pruning. (3) Repeat the second step until the number of branches has increased to the set value. Then we average the predicted results.

### Back propagation neural network analysis

The LASSO-RF overlapping immune cells were included in the multivariate COX analysis to identify significant candidate features. Subsequently, the candidate features are reversely verified through the artificial neural network function input. BPNN is a hierarchical neural network consisting of the input, hidden, and output layer. The algorithm continuously adjusts the network model parameters by back-propagating the calculation error and correcting it simultaneously to maintain a one-to-one correspondence close to the target. BPNN aims to iteratively change the weights between neurons to minimize the error, defined as the squared difference between the expected and actual results of the output node, summed over the training pattern (training dataset) output neuron. BPNN reduces the error between the predicted result and the true output by changing the weight.

### Statistical analysis

The study performed bioinformatics analysis using R v3.6.1 environment. p < 0.05 was considered statistically significant. The regression of K-M and COX relies on the “survival v3.2-3” package. The tROC (time-dependent receiver operating characteristic) and LASSO algorithms are implemented by “timeROC v 0.4” and “glmnet v4.0-2” respectively.

## Results

### Clinical manifestations of FAM83A

**Figure 1:**
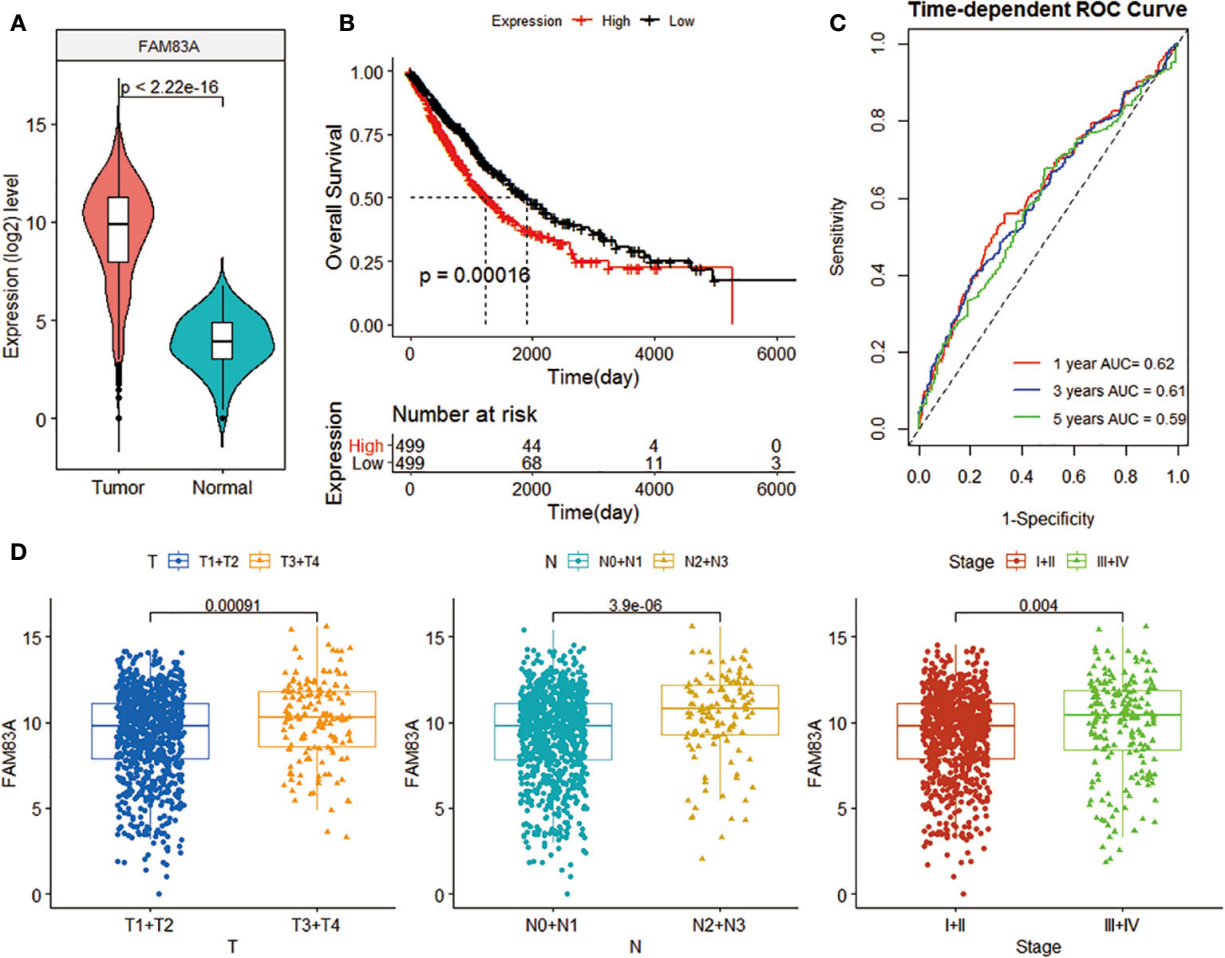
The clinical benefit of FAM83A gene expression level. **(A)** Violin plot of FAM83A expression levels in tumor and normal samples in the TCGA data set. **(B)** K-M curve related to FAM83A expression level and prognosis. The black and red curves refer to the low and high expression sample groups, respectively. **(C)** 1-, 3-, and 5-year ROC curves based on the FAM83A expression. **(D)** Box plot of the correlation between FAM83A expression level and T, N, and Stage.

**Figure 2:**
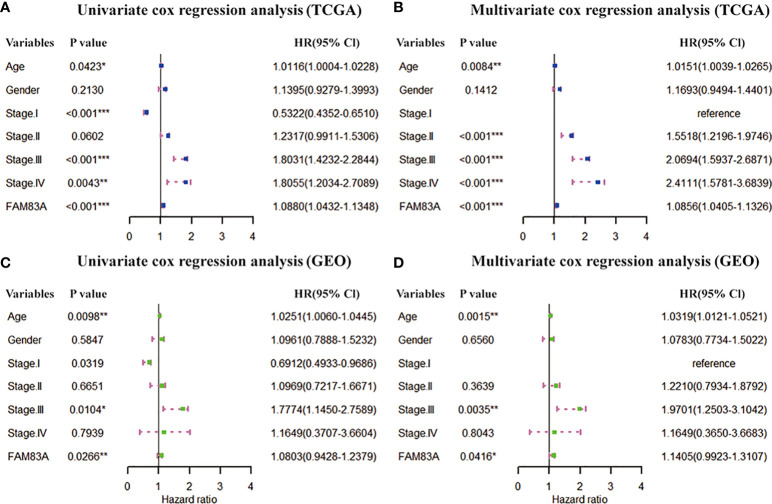
Prognostic independence of FAM83A based on TCGA and GEO datasets. **(A)** Univariate and **(B)** multivariate COX forest plot of FAM83A gene and clinical factors based on the TCGA data set. **(C)** Univariate and **(D)** multivariate COX forest plots of FAM83A and clinical factors based on GEO dataset. (*p<0.05, **p<0.01, ***p<0.001).

**Figure 3:**
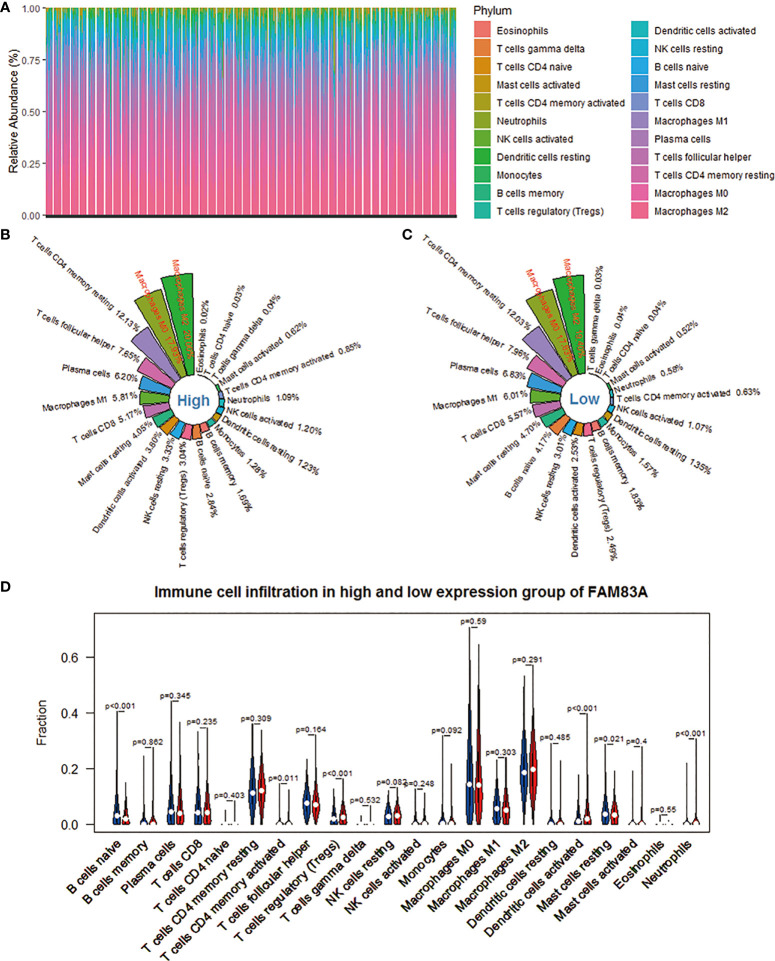
Evaluation of the infiltration level of 22 immune cells in the NSCLC microenvironment based on the CIBERSORT algorithm. **(A)** The percentage abundance of 22 kinds of immune cells in NSCLC samples. **(B)** The distribution of 22 immune cells in the FAM83A high expression group. **(C)** The distribution of 22 immune cells in the FAM83A low expression group. **(D)** Differences in immune infiltration between FAM83A high and low expression groups. Red represents the high expression group, and blue represents the low expression group.

**Figure 4:**
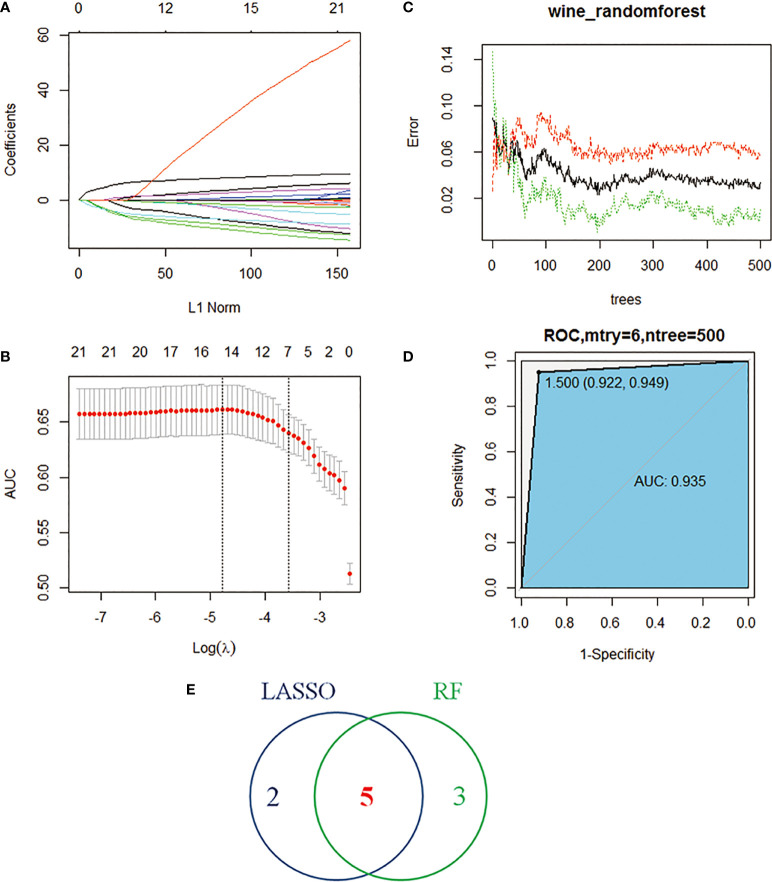
Machine learning identifies key immune cells. **(A)** Distribution of LASSO coefficients for 22 immune cells. **(B)** Penalty plot of 22 immune cells in the LASSO model, error bars represent standard error. **(C)** the error variation of the RF algorithm, red and green represent the error rate of high and low FAM83A expression groups, and black represents the overflow error rate. **(D)** Random forest analysis results **(E)**. Five immune cell types identified by LASSO and RF algorithms.

FAM83A expression in NSCLC was significantly higher than in normal tissues (p < 2.22e-16; **Figure 1A**). The Kaplan-Meier curve, time-dependent ROC curve, T, N, Stage confirmed the clinical performance of FAM83A (**Figures 1B–D**). The survival analysis found that low FAM83A expression substantially improves the patient’s survival (p = 0.00016; **Figure 2B**). In addition, tROC showed that the 1, 3, and 5-year area under curve (AUC) values expressed by FAM83A were 0.62, 0.61, and 0.59, respectively (**Figure 3C**). **Figure 4D** shows that the expression level of FAM83A in the later stages of T (p = 0.00091), N (p = 3.9e-06) and Stage (p = 0.004) was significantly higher than in the early stage.

### The independent prognostic value of FAM83A

The clinical information (age, gender and stage) and FAM83A were included in the Cox regression to estimate the independent prognostic value of FAM83A. Univariate COX regression in the TCGA dataset showed FAM83A (HR = 1.0880, p < 0.001), Age (HR = 1.0116, p = 0.0423) and Stage (stage I, HR = 0.5322, p < 0.001; stage III, HR = 1.8031, p < 0.001; stage IV, HR = 1.8055, p = 0.0043) are prognostic risk factors for NSCLC (**Figure 2A**). The multivariate Cox regression found that FAM83A (HR=1.0856, p < 0.001), Age (HR=1.0151, p = 0.0084) and stage (stage II, HR = 1.5518, p < 0.001; stage III, HR = 2.0694, p < 0.001; stage IV, HR = 2.4111, p < 0.001) is an independent marker for assessing the survival of NSCLC (**Figure 2B**). Similarly, the GSE37745 dataset further confirmed the prognostic independence of FAM83A (**Figures 2C, D**).

### Immune cell infiltration

The CIBERSORT deconvolution algorithm obtains the percentage of infiltration of 22 immune cells in each NSCLC sample. We found that macrophages M2, M0, and T cells CD4 memory resting accounted for a larger proportion (**Figure 3A**). Subsequently, we took the arithmetic mean of the immune cell infiltration rate of 998 NSCLC samples as its infiltration rate in the FAM83A high expression group and low expression group. Each immune cell subtype’s infiltration ratio is the ratio of its population to the total number of 22 immune cells in the immunological microenvironment. The top 5 immune cell subtypes with the highest penetration rate in the FAM83A high expression group are macrophages M2 (20.00%), macrophages M0 (17.93%), T cells CD4 memory resting (12.13%), T cells follicular helper (Tfh) (7.65%), and plasma cells (6.20%) (**Figure 3B**). The top five immune cell subtypes with the highest infiltration rate in the FAM83A low expression group were macrophages M2 (19.40%), macrophages M0 (17.63%), T cells CD4 memory resting (12.03%), Tfh (7.96%) and plasma cells (6.83%) (**Figure 3C**). **Figure 3D** shows 6 differently infiltrating immune cells, namely naive B cells (p < 0.001), T cells CD4 memory activated (p = 0.011), T cells regulatory (Tregs) (p < 0.001), dendritic cells activated (p < 0.001), mast cells resting (p = 0.021) and Neutrophils (p < 0.001).

### Identification of key immune cells

The study performed LASSO and RF analysis on 22 immune cell infiltration. LASSO further reduced the size of 22 immune cells to 7 (**Figures 4A, B**), namely naive B cells, memory B cells, Tregs, T cells CD4 memory activated, Tfh, dendritic cells activated and neutrophils (**Table 1**). RF analysis obtained 8 key immune cells (**Figure 4C**), namely naive B cells, Tregs, T cells CD4 memory resting, macrophages M1, macrophages M2, neutrophils, Tfh and dendritic cells activated (**Table 2**). At the same time, the AUCs of the RF model is 0.935 (**Figure 4D**). **Figure 4E** identifies five overlapping immune cells (naive B cells, Tregs, Tfh, dendritic cells activated and neutrophils).

**Table 1:** LASSO analysis of 7 candidate immune cells.

Immune cells	LASSO Coefficient
B cells naive	1.6275
T cells regulatory (Tregs)	-0.9268
B cells memory	1.3139
T cells CD4 memory activated	-0.2186
Dendritic cells activated	-1.9478
Neutrophils	-0.6484
T cells follicular helper	-1.6101

**Table 2:** RF analysis of 8 candidate immune cells.

Immune cells	MeanDecreaseGini
B cells naive	36.7805
T cells regulatory (Tregs)	36.1051
T.cells CD4 memory resting	34.2748
Macrophages M1	32.4069
Macrophages M2	31.6643
Neutrophils	31.6200
T cells follicular helper	30.5608
Dendritic cells activated	29.3928

### Identification of candidate features

**Figure 5:**
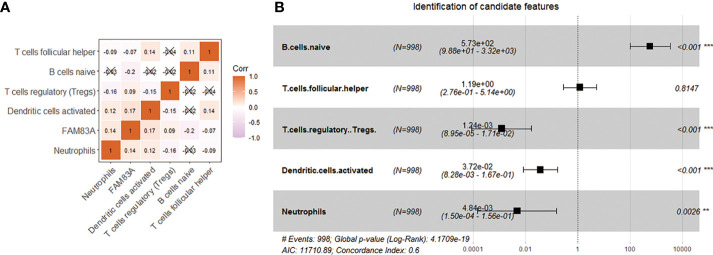
Use correlation analysis to filter features. **(A)** Correlation graph between the infiltration level of 5 immune cells and the expression level of FAM83A gene. **(B)** Five kinds of immune cells and FAM83A related forest plot. **p<0.01, ***p<0.001.

FAM83A expression is negatively correlated with naive B.cells. (Cor = -0.20) and Tfh (Cor = -0.07), and significantly positively correlated with DCs activated (Cor = 0.17), Tregs (Cor = 0.09) and neutrophils (Cor = 0.14) (**Figure 5A**). We further included five immune characteristics in the multivariate COX regression. The results showed that naive B cells (p < 0.001), Tregs (p < 0.001), dendritic cells activated (p < 0.001) and neutrophils (p = 0.0026) are the key immune landscapes associated with FAM83A (**Figure 5B**). Therefore, FAM83A associated 4 types of immune cells served as candidate features.

### BPNN verification

**Figure 6:**
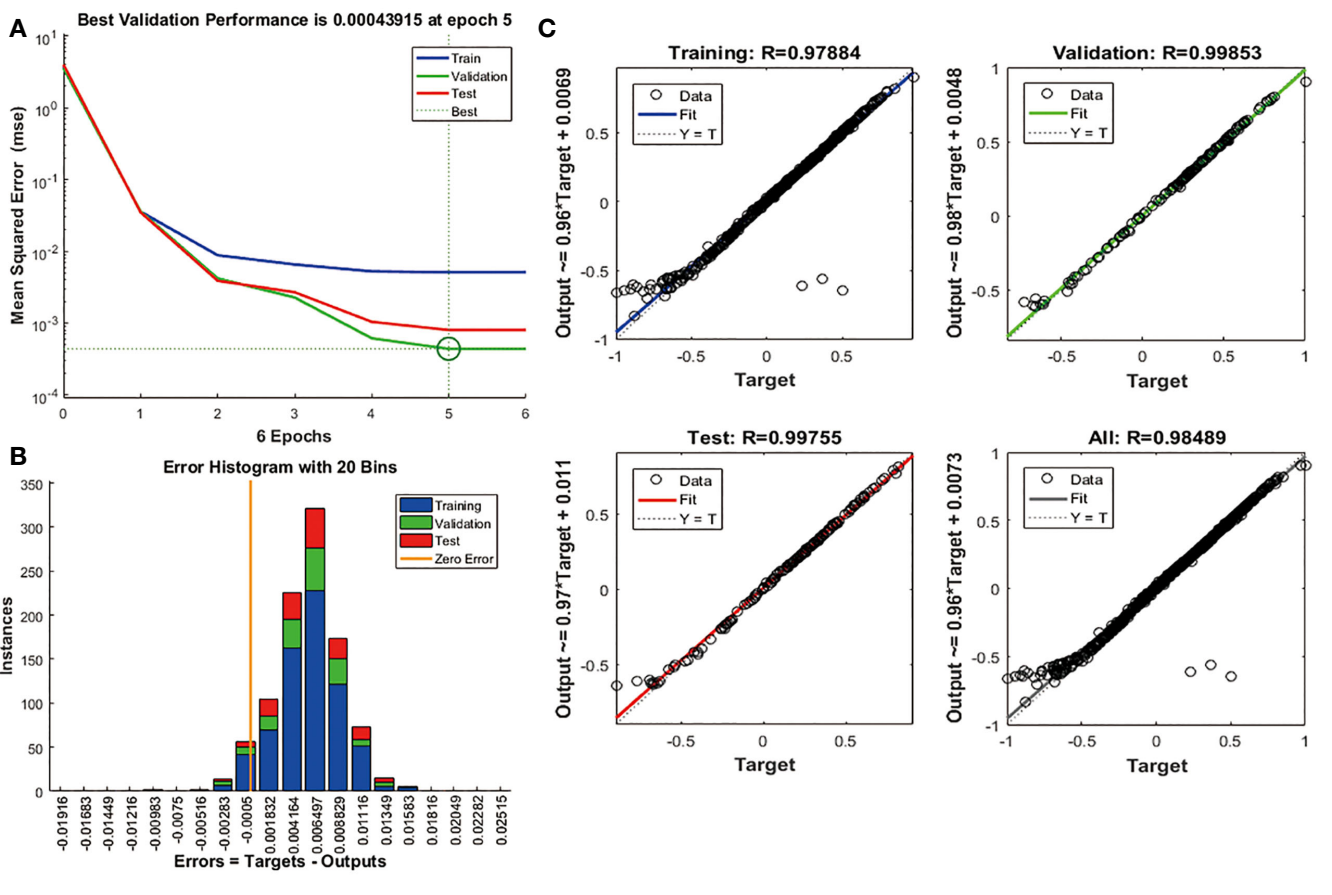
Validation of candidate features based on BPNN algorithm. **(A)** The change curve of MSE and epochs of the BPNN model. **(B)** The error distribution bar graph of the BPNN model. **(C)** Linear regression graph of BPNN model.

We use candidate features as the input of BPNN to further verify the machine learning results. The number of hidden layers set by BPNN is 2, and the number of nodes is 10. The FAM83A expression acted as the output of the algorithm. 70% of the data served as the training data set, and the other 30% validates and tests the neural network. The study executed the algorithm based on mean square error (MSE). The result proves that the MSE is 0.00043915, and the best performance of the BPNN model appeared at 5 epochs (**Figure 6A**). **Figure 6B** shows that the prediction error range is -0.01916 to 0.02515. In addition, we also found that the training set (R = 0.97884), the validation set (R = 0.99853), and the test set (R = 0.99755) have high regression values (**Figure 6C**), indicating a small prediction bias of the BPNN model. These results suggest that the candidate features are significantly related to FAM83A expression.

### Immune cells associated with FAM83A

**Figure 7:**
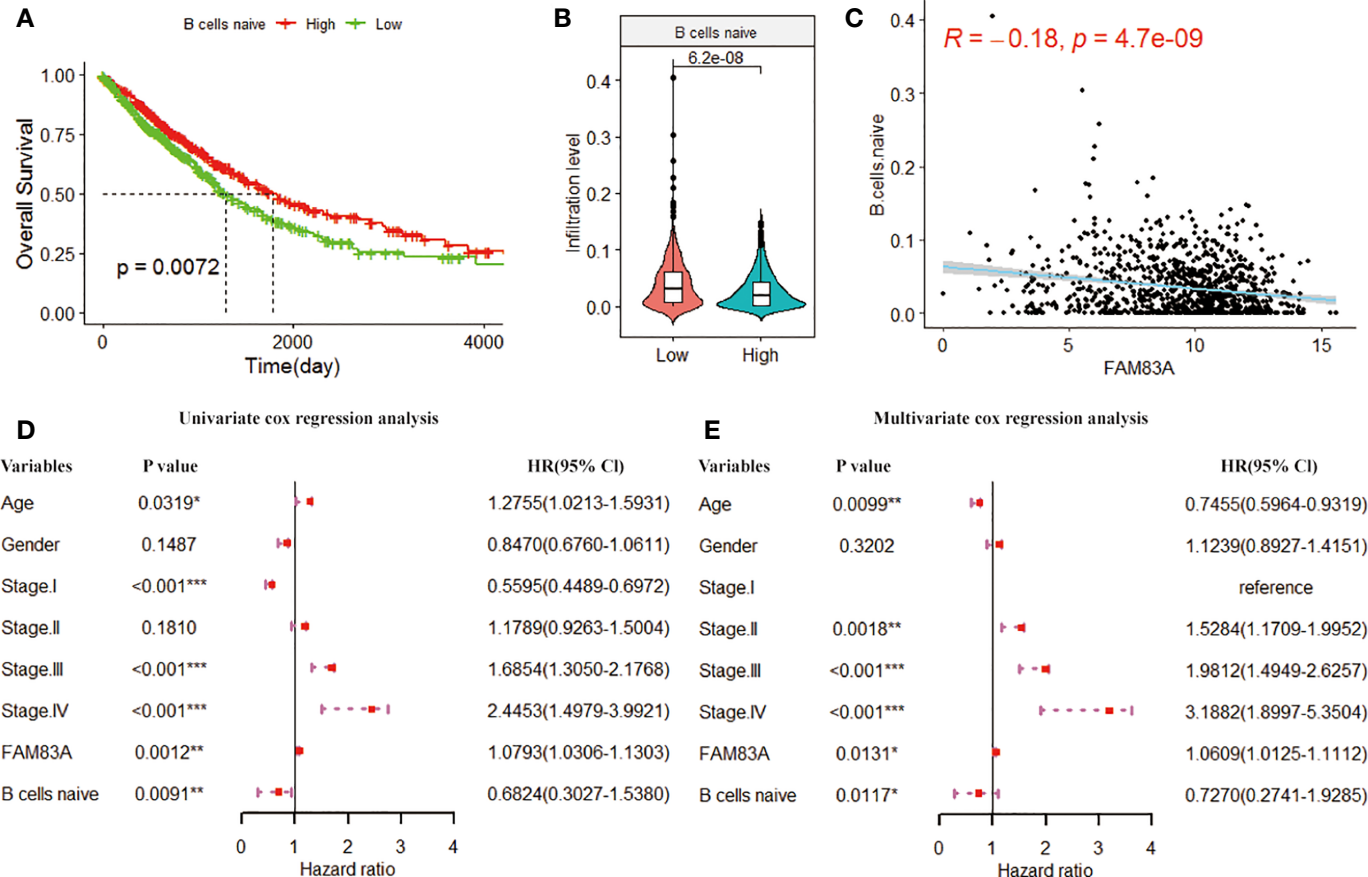
The K-M curve of **(A)** naive B cells infiltration level and overall survival. The green and red curves represent the sample group with low expression and high infiltration level, respectively. **(B)** Violin plot of naive B cell infiltration in FAM83A high and low expression groups. **(C)** Scatter plot of the correlation between the infiltration level of naive B cells and the FAM83A expression. **(D)** Univariate cox forest plots related to the prognosis of clinical factors. **(E)**. Multivariate cox forest plots related to the prognosis of clinical factors. (*p<0.05, **p<0.01, ***p<0.001).

The study further investigates the prognostic ability of immune cells associated with FAM83A. We performed K-M analysis on FAM83A associated immune cells and found that the low infiltration of naive B cells was significantly associated with the poor prognosis of NSCLC patients (p = 0.0072; **Figure 7A**). However, Tregs, dendritic cells activated and neutrophils have no correlation with the overall survival of NSCLC patients (**Figure S1**). Naive B cells infiltration in the low expression group was significantly higher than in the FAM83A high expression group (p = 6.2e-08; **Figure 7B**). **Figure 7C** shows that the naive B cells infiltration is significantly correlated with the FAM83A expression (Cor = -0.18, p = 4.7e-09). Then we included naive B cells, FAM83A and clinical information into the Cox regression analysis after excluding samples with an immune score of 0. Univariate and multivariate analysis showed that FAM83A and naive B cells are risk factors for the prognosis of NSCLC (p < 0.05, **Figure 7D, E**). Therefore, we confirmed that the abnormal infiltration of naive B cells associated with FAM83A is a key factor in predicting the prognosis of NSCLC patients.

### Nomogram analysis

**Figure 8:**
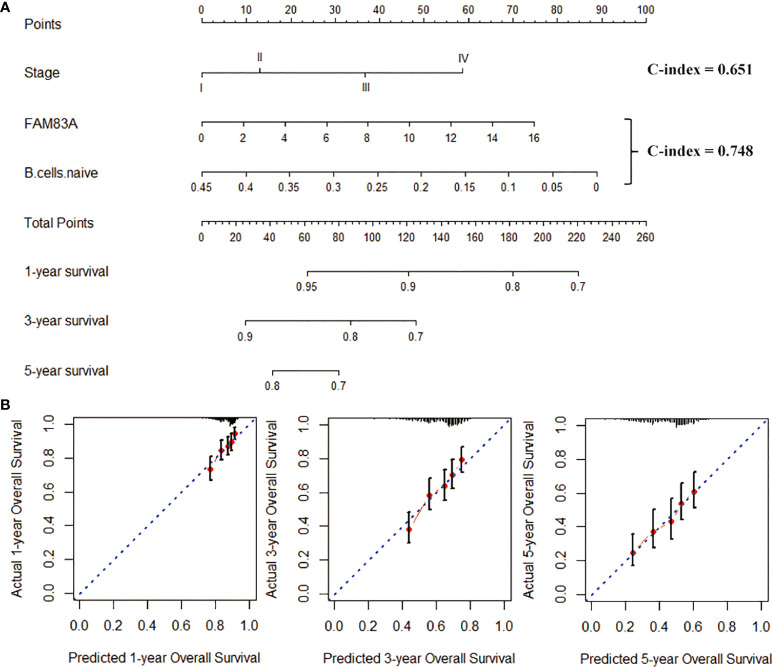
Construction of the nomogram graph. **(A)** Stage, FAM83A and naive B cells survival rate prediction model nomogram. **(B)** The nomogram is a line graph of the consistency between the predicted and actual survival rates of 1-, 3-, and 5-year survival rates. The horizontal and the vertical axis represents the predicted and the actual survival rate, respectively.

We included naive B cells and FAM83A in the nomogram construction due to the excellent prognostic ability. The nomogram created by combining naive B cells and FAM83A (C-index = 0.748; **Figure 8A**) has better prognostic predictive ability than Stage (C-index = 0.651). And the nomogram matches the best prediction performance (**Figure 8B**).

## Discussion

The 5-year survival rate of advanced NSCLC is 5-15% (15). As immunotherapy has made significant breakthroughs in NSCLC, the human immune system recruits and activates T cells to recognize and eliminate cancer cells (16). However, not every patient responds to this treatment. Therefore, clarifying and finding new immune-related therapeutic targets may provide more clues for targeted therapy of NSCLC. Studies have found (17, 18) that FAM83 family members A, B, and D have carcinogenic potential. The expression of FAM83A is also related to the growth rate of tumors. The expression of the endogenous FAM83A and the increase in DNA copy number make the surviving tumor cells resistant to treatment (19).

FAM83 family genes activate the MEK/ERK signaling pathway to affect the occurrence and development of tumors (20). FAM83A has long been identified as a tumor-specific biomarker and is highly expressed in nearly half of lung cancer tissue samples. FAM83A promotes the progression of NSCLC through ERK and PI3K/Akt/mTOR signaling pathways (9). Zheng et al. (21) found that FAM83A enhances the proliferation and invasion of NSCLC cells by modulating the Wnt and Hippo signaling pathways, as well as the EMT process. FAM83A is highly expressed in NSCLC, related to advanced TNM staging and poor prognosis, consistent with the study. The study analyzed the relationship between the FAM83A expression and the prognosis, survival rate, tumor stage, and lymph node metastasis of NSCLC patients through TCGA data. NSCLC patients with high FAM83A expression have a low survival rate that is significantly related to lymph node metastases and NSCLC clinical stage. Zhang et al. (22) proved that the FAM83A expression in advanced NSCLC tumors (stages α to α) is higher than that in early stage NSCLC tumors (stagesαtoα). This is consistent with the significant correlation between the FAM83Aexpression and the NSCLC stage in this study. The above analysis suggests that FAM83A may be a prognostic biomarker for NSCLC.

We evaluated the level of immune cell infiltration based on the CIBERSORT algorithm. Among them, macrophages M2, macrophages M0 and T cells CD4 memory resting accounted for a relatively large proportion of NSCLC. Macrophages are highly varied and can be classified into M1 and M2 subtypes as they progress from the M0 stage, through local microenvironmental stimulation (23). Macrophages M2 suppressed inflammation and reduced the body’s immune response by secreting inhibitors to promote tumor metastasis (24). In addition, Jin et al. (25) found that the heterogeneity of immunotherapy in NSCLC patients was significantly associated with abnormal infiltration of T cells CD4 memory resting.

The LASSO and RF algorithms helped us identify five immune infiltrating cells significantly related to NSCLC patients. Studies have found that the expansion of Tregs hinders tumor immunotherapy, leading to immunotherapy failure (26). Furthermore, studies have found high Tregs infiltration is associated with poor prognosis of NSCLC patients (27, 28). Guo et al. (29) confirmed that the infiltration rate of Tfh in advanced-stage patients is higher than that in early-stage patients. Tfh may be an important prognostic biomarker for evaluating the adverse clinical outcome of NSCLC patients (30). Li et al. (31) found that DCs activated immunotherapy has a good effect on treating non-small cell lung cancer. In addition, neutrophil content can predict lymphocyte depletion and failure of anti-PD-1 therapy in NSCLC (32).

The LASSO algorithm has the function of dimensionality reduction, which can directly reduce the number of features and make the feature selection. When the features have strong semantics, it is better to use LASSO, and the subsequent analysis will be more interpretable. The random forest is an ensemble learning algorithm that can calculate the features that contribute the most to the final result. BPNN can combine data, and the final result is an inexplicable model that can only realize input data and make predictions. Therefore, the combination of LASSO, random forest, and BPNN can reasonably achieve a comprehensive dimensionality reduction, feature selection, and interpretability analysis.

Cox regression, BPNN algorithm and K-M analysis demonstrated that the naive B cells’ low infiltration was significantly related to the poor prognosis of NSCLC patients, confirmed by Chen et al. (33). Naive B cells participated in the immunosuppressive process and promoted the development of lung cancer (34). Mechanism 1: the high infiltration of naive B cells promotes the immune activity of patients with lung cancer (35). Mechanism 2: naive B cells with low infiltration in the lung cancer microenvironment directly promotes the proliferation of lung cancer cells (36). In addition, we confirmed that the naive B cells infiltration was negatively correlated with the FAM83A expression. At the same time, FAM83A and naive B cells are independent risk factors for the prognosis of NSCLC patients. Therefore, abnormal infiltration of naive B cells associated with FAM83A is a key factor in predicting the NSCLC patients’ prognosis.

Compared with other clinical features, FAM83A and naive B cells showed good prognostic independence. Interestingly, Cox regression analysis shows the powerful predictive ability of stage. The stage provided effective prognostic diagnosis and appropriate treatment guidance (37). FAM83A and naive B cells prognostic characteristics (C-index = 0.748) have more advantages in survival prediction than Stage (C-index = 0.651). Then, the nomogram constructed by combining FAM83A and naive B cells further improved the accuracy of survival prediction.

## Conclusion

The study found that FAM83A has excellent prognostic ability based on various machine learning algorithms. Thus, FAM83A may serve as a potential prognostic marker for NSCLC. Subsequently, we applied the SVM-based CIBERSORT algorithm to assess the immune cell components of the NSCLC microenvironment. We used three machine learning algorithms to identify naive B cells significantly related to the survival of NSCLC patients. In addition, the nomograms of naive B cells and FAM83A better predict the overall survival rate of NSCLC than in the traditional stage. Therefore, the abnormal infiltration of naive B cells associated with FAM83A may be a critical factor in predicting the prognosis of NSCLC patients. Still, it needs to be further verified by clinical trials.

## Data availability statement

The original contributions presented in the study are included in the article/**Supplementary Material**. Further inquiries can be directed to the corresponding authors.

## Author contributions

PT wrote the manuscript. ZW designed the study. XL and LC contributed to the data collection. MZ and ZX contributed to data analysis and figure preparation. The authors read and approved the final manuscript.

## Funding

The study was supported by Natural Science Foundation of Xiaogan (Grant Number: XGKJ2021010024).

## Conflict of interest

The authors declare that the research was conducted in the absence of any commercial or financial relationships that could be construed as a potential conflict of interest.

## Publisher’s note

All claims expressed in this article are solely those of the authors and do not necessarily represent those of their affiliated organizations, or those of the publisher, the editors and the reviewers. Any product that may be evaluated in this article, or claim that may be made by its manufacturer, is not guaranteed or endorsed by the publisher.

## Abbreviations

AUC, area under curve; BP, back propagation neural network; EGFR-TKI, epidermal growth factor receptor-tyrosine kinase inhibitor; FAM83A, family with sequence similarity 83 member A; GEO, gene expression omnibus; K-M, Kaplan-Meier; LASSO, least absolute shrinkage and selection operator; LUAD, lung adenocarcinoma; LUSC, lung squamous cell carcinoma; MSE, mean square error; NSCLC, non-small cell lung cancer; RF, random forest; ROC, receiver operating characteristic; SVM, support vector machine; TCGA, the cancer genome atlas; Tfh, T cells follicular helper; Tregs, T cells regulatory.
