# Supplementary figures and images for: Machine learning and BP neural network revealed abnormal B cell infiltration predicts the survival of lung cancer patients
Source: Front Oncol. 2022 Oct 11;12:882018. doi: 10.3389/fonc.2022.882018 (PMC9592816; doi:10.3389/fonc.2022.882018)

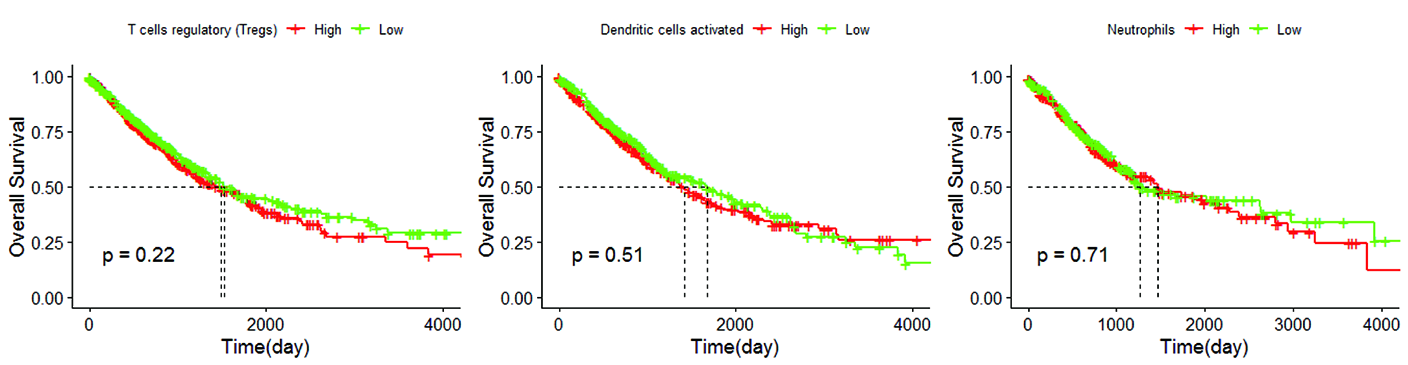

Supplement: Supplementary file 1 [file Image_1.tif]
